# Recruiting Eligible and Interested Study Participants With Cognitive Impairment

**DOI:** 10.1093/geroni/igab046.121

**Published:** 2021-12-17

**Authors:** Meghan Mattos, Jennifer Lingler

## Abstract

Recruiting and enrolling older adults with cognitive impairment is challenging under the best of circumstances. This symposium will begin with an introduction to best practices for recruitment of older adults living with cognitive impairment, followed by four presentations describing recruitment successes and challenges across multiple settings. The first presentation describes COVID-19 pandemic-related factors that have influenced recruitment and enrollment of older adults with cognitive impairment in an intervention study of a physical activity smartphone app. Strategies and procedural alterations to facilitate achievement of enrollment goals for technology-based interventions are discussed. The second presentation describes researchers’ recruiting experiences with older adults with mild cognitive impairment (oaMCI)-care partner dyads for a pilot, platform trial of biopsychosocial interventions. There were differences in study disinterest between oaMCI and study partners that may require specialized communication messaging and strategies for dyad engagement. The third presentation features recruitment adaptations for an Internet-delivered behavioral intervention study with oaMCI and insomnia. Anticipated concerns of oaMCI using technology or accessing the Internet were not significant barriers to recruitment, while fewer oaMCI endorsed sleep concerns than expected. The last presentation demonstrates the potential for telephone-based outreach to increase dementia knowledge and cognitive risk. Working with faith-based health educators to reach rural, ethnically-diverse older adults, researchers will describe how to promote inclusivity and successfully recruit oaMCI within the community. Presenters and participants are encouraged to dialogue on how recruitment and retention barriers may be avoided as well as to share success stories from their own research with oaMCI.

